# Extrinsic and Intrinsic Regulation of Axon Regeneration by MicroRNAs after Spinal Cord Injury

**DOI:** 10.1155/2016/1279051

**Published:** 2016-10-13

**Authors:** Ping Li, Zhao-Qian Teng, Chang-Mei Liu

**Affiliations:** ^1^State Key Laboratory of Stem Cell and Reproductive Biology, Institute of Zoology, Chinese Academy of Sciences, Beijing 100101, China; ^2^Department of Spine Surgery, Xiangya Hospital, Central South University, Changsha 410008, China; ^3^Savaid Medical School, University of Chinese Academy of Sciences, Beijing 100049, China

## Abstract

Spinal cord injury is a devastating disease which disrupts the connections between the brain and spinal cord, often resulting in the loss of sensory and motor function below the lesion site. Most injured neurons fail to regenerate in the central nervous system after injury. Multiple intrinsic and extrinsic factors contribute to the general failure of axonal regeneration after injury. MicroRNAs can modulate multiple genes' expression and are tightly controlled during nerve development or the injury process. Evidence has demonstrated that microRNAs and their signaling pathways play important roles in mediating axon regeneration and glial scar formation after spinal cord injury. This article reviews the role and mechanism of differentially expressed microRNAs in regulating axon regeneration and glial scar formation after spinal cord injury, as well as their therapeutic potential for promoting axonal regeneration and repair of the injured spinal cord.

## 1. Introduction

Spinal cord injury (SCI), one of the most severe injuries, causes the death of many kinds of cells such as neurons, oligodendrocytes, and astrocytes, in which extensive loss of sensory and motor functions occurs below the injury site [1]. Two different mechanisms have been proposed for the pathogenesis of SCI: a primary mechanical injury and a secondary injury induced by multiple biological processes, including ongoing apoptosis, inflammation, excitotoxicity, and extensive demyelination of axons [1, 2]. Prior studies have suggested that, unlike the peripheral nervous system (PNS), the adult mammalian central nervous system (CNS) has limited axon regeneration ability after SCI, predominantly due to the inability of neurons to regenerate axons through the inhibitory milieu of the glial scar and injured spinal cord lesion [3], which impede the functional recovery after trauma. Numerous lines of evidence suggest that axonal regeneration and functional recovery can be influenced by intrinsic and extrinsic factors, and the regeneration and recovery of SCI is a complex multicellular response, with multiple cell types having numerous roles in distinct regions of the nerve.

The axonal regeneration ability mainly depends on extrinsic environment inhibitory elements and neuronal intrinsic regenerative potential [3–7]. The injured adult CNS is a nonpermissive environment for axon regeneration due to the abundance of inhibitory proteins and glycoproteins [5]. Moreover, intrinsic neuronal mechanisms initiating a growth program are also very limited in injured adult CNS neurons [6]. Because of the failure of CNS axons to spontaneously regenerate, sensory, motor, autonomic, or cognitive deficits resulting from CNS injury are often permanent [8].

Epigenetic regulation plays a pivotal role in various physiological and pathological processes by regulating gene expression, such as apoptosis, proliferation, hematopoiesis, differentiation, regeneration, and development [9–14]. MicroRNAs are a big class of critical epigenetic regulation factors, and about 77% of the identified mature noncoding microRNAs have been discovered in the rodent spinal cord [15]. MicroRNAs play important roles in regulating the process of neuronal plasticity, neuronal degeneration, axonal regeneration, and remyelination via translational repression or leading to mRNA degradation [16–19]. Alterations in the expression of many genes during spinal cord process have been shown to play vital roles in the pathogenesis of secondary SCI or axon regeneration [20]. Evidence has shown that plenty of microRNAs changed dramatically [21], which suggests that microRNAs are involved in the pathogenesis of SCI. In this review, we summarize the dysregulated microRNAs after SCI and their roles in mediating glial scar formation and intrinsic and extrinsic axon degeneration. We also discuss the microRNA-based therapeutic strategies for promoting axonal regeneration after SCI.

## 2. Altered MicroRNAs Expression following SCI

More and more evidences have demonstrated that microRNAs are highly abundant in the spinal cord and are dysregulated following SCI (Table 1). Actually, a total of 3,361 microRNAs have been identified to be expressed in the spinal cord of adult rats [22], and among them 60 microRNAs are reported to be dysregulated at different time-points after SCI. In another study, 32 microRNAs, including miR-124, miR-129, and miR-1, are significantly downregulated, but miR-21 is significantly upregulated in the injury sites of contused rat spinal cords [23]. Similar observations are made in another microarray study of a rat contusive SCI model, in which 343 microRNAs are found to be modulated following injury, and most of them are downregulated at day 7 after injury compared with baseline [24]. Interestingly, in a mouse SCI model, miR-223 expression is maintained upregulated until 3 days after SCI. However, miR-124a expression is significantly decreased from day 1 to day 7 after injury [21]. In consistency with these findings, several other microRNAs profiling studies also suggest that abnormal expression of microRNAs may contribute to the pathogenesis of SCI [25–27].

Bioinformatic analysis indicates that the potential targets of dysregulated microRNAs after SCI include genes that are involved in the pathogenesis of SCI, including inflammation, oxidation, apoptosis, and neuroplasticity. Thus, the dysregulated microRNAs are considered as potential targets for therapeutic interventions following SCI [15]. Among those dysregulated microRNAs after SCI, microRNA-21 and microRNA-146a have been evidenced to promote neurological function through reducing apoptosis and astrocytes hypertrophic response to injury [28, 29]. miR-181a, miR-411, miR-99a, miR-133b, and miR-15b can enhance the inflammation response to injury [29–32]. Meanwhile, miR-221, miR-126, miR-223, and Let-7a repress inflammation by targeting several proinflammatory genes [33–35]. In addition, several other studies show that microRNAs can also regulate endogenous antioxidant systems after SCI [36–38] and modulate remyelination via targeting superoxide dismutase (SOD), the antioxidant enzyme defense system [39]. Therefore, microRNAs have been suggested as biomarkers as well as therapeutic targets of the pathological process of SCI [40].

## 3. MicroRNAs and Glial Scar Formation

SCI induces a chronic wound state that undergoes expansion and maintained demyelination resulting in impaired recovery and progressive tissue degeneration [41, 42]. Maladaptive inflammation, specifically the activation of glial cells, is likely a contributor. Astrocyte is the most abundant cell type in the CNS comprising over 50% of total glial cell number [43]. After SCI, astrocytes can be activated by undergoing proliferation (astrocytosis) and hypertrophy (astrogliosis). A large number of studies report that the astrogliosis can influence myelin debris secretion and scar tissue formation via regulating inflammation response and astrocytes hyperplastic [43–46]. Astrogliosis, myelin debris, and scar tissue play the decisive role in the process of axon regeneration failure after CNS damage, which may be essential for wound repair, but also inhibit axonal regrowth [47, 48]. Hence, a potential treatment to injuries in the CNS could be controlling glial scar formation by modulating the proliferation and hypertrophy extents of astrocytes surrounding the injured site.

It is also possible to promote axon regeneration after SCI by targeting astrogliosis, which has been verified by some researches. For example, blocking the reactive gliosis after SCI can enhance functional recovery and promote axon regrowth to some extent [49]. Astrogliosis inhibition can also result in increased axon density within the lesion site. Regardless of neuron-astrocyte coculture or neurite guidance spot assay, the astrogliosis usually shows inhibitory effects on neuron growth [49]. Surprisingly, a recent study shows that contrary to the prevailing dogma astrocyte scar formation aids rather than prevents CNS axon regeneration. Using three genetically targeted loss-of-function manipulations in adult mice, researchers provide evidence showing that preventing astrocyte scar formation, attenuating scar-forming astrocytes, or ablating chronic astrocytic scars all failed to result in spontaneous regrowth of transected corticospinal, sensory, or serotonergic axons through severe SCI lesions [50].

Astrogliosis is regulated by several well-known pathways, such as cAMP, STAT3, NF-kB, Rho-kinase, JNK, and mTOR [51–54]. For example, astrocytic reactivity is dependent on RhoA signaling pathway activity and the astrocytic reactivity can be reduced by targeting RhoA [50]. Injury-induced cytokines, such as ciliary neurotrophic factor (CNTF), interleukin-6, transforming growth factor alpha, fibroblastic growth factor-2, and epidermal growth factor, have been reported to enhance astrocyte activity, attributed to glial scar formation [55]. In addition, PTEN might have a role in early stage of reactive astrogliosis in vivo via P13K/Akt/mTOR signaling pathway [53], and Jagged-1 can regulate the activation of astrocytes via modulation of NF-kB and JAK/STAT/SOCS3 signaling pathway [56, 57]. In recent years, new evidences also suggest that microRNAs are involved in the major signaling pathway participating in astrogliosis. For example, microRNA-582 and microRNA-590 target NF-kB signaling pathway; miR-205 inhibits tumor growth by targeting cAMP; miR-146a, miR-133b, and miR-124 can directly regulate RhoA signaling pathway [35, 58]. Although direct evidences are lacking, these findings suggest that these microRNAs might also be involved in the process or recovery of SCI through regulating these important pathways.

Several other microRNAs might also play critical roles in astrogliosis and glial scar formation. Overexpression of miR-21 in wild-type serum-derived astrocytes causes a dramatic reduction in cell size accompanied by reduction in glial fibrillary acidic protein (GFAP) levels. Conditional ablation of BMPR1a from GFAP-expressing cells leads to defective astrocytic hypertrophy, increased infiltration by inflammatory cells, and reduced axon density. BMPR1b knockout mice have an attenuated glial scar in the chronic stages following injury. Further analysis demonstrates that BMPR1a and BMPR1b exert opposing effects on the posttranscriptional regulation of astrocytic miR-21. Hence, targeting miR-21 has been suggested as a therapeutic approach for manipulating gliosis and enhancing functional outcomes after SCI [59]. miR-145, a microRNA enriched in spinal neurons and astrocytes, is significantly downregulated after SCI [39]. Overexpression of miR-145 in astrocytes by a lentivirus-mediated pre-microRNA delivery system with GFAP promoter at the spinal cord lesion site reduces the density of astrocytes at the lesion border of the injured spinal cord [39]. In parallel, overexpression of miR-145 decreases the size of astrocytes and the number of related cell processes, as well as cell proliferation and migration. These findings suggest that the downregulation of miR-145 in astrocytes is a critical factor inducing astrogliosis after SCI [39]. In contrary to miR-145, miR-125b is upregulated in interleukin-6- (IL-6-) stressed normal human astrocytes (NHA), a treatment known to induce astrogliosis [48]. In vitro, anti-miR-125b treatment in IL-6-stressed NHA cultures attenuates glial cell proliferation and increases the expression of the cyclin-dependent kinase inhibitor 2A (CDKN2A), a negative regulator of cell growth. Interestingly, a strong positive correlation between miR-125b abundance and GFAP/vimentin also exists in CNS of advanced Alzheimer's disease and Down's syndrome patients [48]. Astrocyte enriched miR-181a is increased in vulnerable regions and decreased in ischemia-resistant areas [60]. Antagomir to miR-181a can reduce infarct size in focal ischemia [61], which suggests that modulation of miR-181 may be a novel therapeutic intervention for injuries in the CNS.

## 4. MicroRNAs and Extrinsic Determinants of Axon Regeneration

Extrinsic barrier mechanisms for injured axons of CNS mainly include the CNS myelin in the injury milieu and the growth inhibitory molecules in the glial scar, and extrinsic inhibitory elements are mainly distributed in oligodendrocytes, astrocytes, microglia, and fibroblast. After injury, these cells can be activated and increased. Then the large number of myelin-associated inhibitors (MAIs) or other inhibitory moles would be released by these cells and form the nonpermissive environment to impede axon regeneration and/or sprouting. Three myelin-associated proteins, namely, myelin-associated glycoprotein (MAG), Nogo-A, and oligodendrocyte myelin glycoprotein (OMgp), and various chondroitin sulfate proteoglycans (CSPGs), like NG2 or Versican, semaphorins, and ephrins, have been identified to be main molecular obstacles to axon regeneration [5]. These inhibitory molecules can be overexpressed in the microenvironment after SCI. A wide number of studies indicate that MAG can inhibit or promote neurite outgrowth depending on the developmental status of the neuron and other factors [4, 62, 63], which provides the potential to improve the neurological function after SCI via promoting axon sprouting or axon regeneration through modulating these extrinsic factors. How to decrease MAIs and other inhibitors and how to impede hostile environment for axon growth after SCI are remaining as challenge questions that desperately need to be solved in this research field.

**Figure 1:**
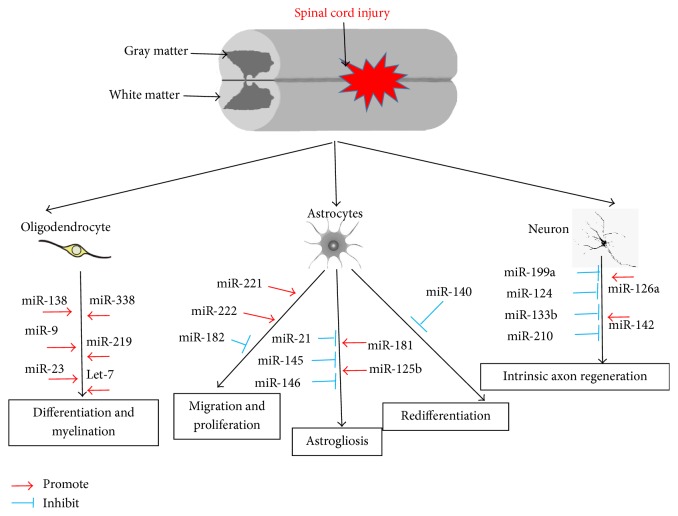
MicroRNAs in the pathogenesis of SCI. SCI triggers a series of pathophysiological responses in the spinal cord, including axon myelination, astrocyte activation, and intrinsic axon regeneration. The activation and inhibition of microRNAs are indicated by a red arrow line and a blue T-shape line, respectively.

MicroRNAs are essential for the development of astrocytes, and astrogliogenesis can be completely blocked by inhibiting microRNA genesis in the spinal cord [64]. For example, miR-130b, miR-21, miR-146a, miR-155, miR-22, miR-622, and miR-145 can regulate astrocytes proliferation, activation, terminal differentiation, and astrocyte-related inflammation [39, 64, 65]. Among these microRNAs, miR-21 is the most well studied (Figure 1) in SCI model [23]. By using transgenic mice in which miR-21 is either overexpressed or inhibited specifically in astrocytes, researchers tested the functions of miR-21 in response to SCI. miR-21 is expressed at low levels in the uninjured spinal, and neither overexpression of miR-21 nor the miR-21 sponge produces observable phenotypic changes in astrocytes in uninjured spinal cords [66]. miR-21 overexpression in astrocytes attenuates the beneficial hypertrophic response, whereas inhibiting the microRNA augments it, suggesting that miR-21 has a significant role in regulating astrocytic hypertrophy and glial scar progression. Inhibition of miR-21 function in astrocytes increases axon density within the lesion site [66], which suggests that miR-21 might be a potential molecular target for manipulating gliosis and enhancing functional outcome after SCI.

A recent study shows that axonal microRNAs regulate axonal growth by modulating local protein composition [67]. In cultured cortical neurons, axonal application of CSPGs inhibits axonal growth and alters axonal microRNA expression profiles, whereas elevation of axonal cyclic guanosine monophosphate (cGMP) levels by axonal application of sildenafil reversed the effect of CSPGs on inhibition of axonal growth and on microRNA expression profiles. These data demonstrate that axonal microRNAs might play an important role in mediating the inhibitory action of CSPGs on axonal growth [67]. miR-146a, a glial-enriched microRNA [68, 69], is reported to target superoxide dismutase (SOD) 2, an endogenous mitochondrial antioxidant enzyme, and regulate cell viability in H_2_O_2_ treated PC12 cells [70]. Another study shows that modulation of miR-146a expression by transfection of astrocytes with anti-miR146a or mimic regulated not only the expression levels of downstream targets of miR-146a (IRAK-1, IRAK-2, and TRAF-6), but also the expression of IL-6 and COX-2 and several cytokines such as IL-6 and TNF-*α*. These observations indicate that in response to inflammatory cues miR-146a is induced as a negative-feedback regulator of the astrocyte-mediated inflammatory response [71]. Moreover, miR-146a also directly targets some astrocyte-specific mRNAs, such as Nlgn1, Nova1, and Syt1 to induce neural stem cell to differentiate into astrocytes [68]. Interestingly, miR-146a has the opposite effect on proneuronal differentiation by targeting neuroligin 1 (Nlgn1) [70]. These data suggest that miR-146a might provide novel clues for modulating axon regeneration through targeting astrocytes.

A regeneration-permissive environment after SCI has been created by precisely regulating miR-125b expression levels in the regeneration-competent axolotl salamander (*Ambystoma mexicanum*), versus the regeneration-incompetent rat [72]. A single dose of miR-125b targets multiple pathways that improve functional recovery after complete transection of the spinal cord. This comparative study offers the first substantial translation of new molecular insights aimed at defining a new biological understanding of major mammalian pathways and new avenues for the development of innovative treatments for human spinal cord injuries [72]. It will be interesting to explore the roles and molecular network of other astrocyte-enriched microRNAs in spinal cord models in axon regeneration in the future research.

It is well known that damage of myelin membranes and failure of remyelination after nerve injury can disrupt neural signals, leading to nerve degeneration. Remyelination has been demonstrated in animal models to be mediated by oligodendrocyte progenitor cells (OPCs), which migrate into the lesion, proliferate, and differentiate into mature OLs and then ensheathe the demyelinated axons. Dicer1 deletion leads to a substantial increase of OPC proliferation and a drastic reduction in myelination, suggesting that microRNAs are required for normal OPC cell cycle exit and differentiation [73, 74]. Actually, miR-219, miR-338, and miR-17–92 are enriched in human white matter and highly expressed in acutely isolated human OLs. In addition, both rodent and human OLs express high levels of closely related microRNAs (miR-219-1-3p, miR-219-2-3p, miR-1250, miR-657, miR-3065-5p, and miR-3065-3p). High expression of microRNAs in OLs suggests that they may regulate myelination program [75]. In support of this, a recently published study suggests that the process of precursor cell transit into mature myelinating OLs is modulated by miR-17–92, miR-199a-5p, and miR-145 [76]. Indeed, miR-219, miR-138, and miR-338 are robustly upregulated upon OPC differentiation, and miR-219 is necessary and sufficient to promote OPC differentiation [73]. In another study, miR-219 is found to be enriched in young and environmental enrichment (EE) serum-derived exosomes, which is necessary and sufficient for production of myelinating oligodendrocytes by reducing the expression of inhibitory regulators of differentiation [77]. miR-23 is abundantly expressed in OLs and involved in oligodendrocyte differentiation, myelin synthesis, maintenance, and proper myelin folding [78, 79]. The overexpression of miR-23a in transgene (TG) mice led to 50%, 80%, and 35% increases in MBP, CNP, and MAG levels, respectively, in the corpus callosum as compared to wild-type mice [78]. The phosphatase and tensin homologue/phosphatidylinositol trisphosphate kinase/Akt/mammalian targets of rapamycin pathway are then identified as downstream targets of miR-23a. Hence, miR-23 treatment could be employed to elevate the expression levels of these myelin genes in the local OLs, and thus facilitating their remyelination process after nerve injury in the CNS [78]. In the PNS, let-7 is found to be abundant during PNS myelination, and its expression levels are inversely correlated to the expression of Lin28B, an antagonist of let-7 accumulation. Sustained expression of Lin28B and consequently reduced levels of let-7 results in a failure of Schwann cell myelination in transgenic mouse models and in cell cultures. Let-7 promotes expression of the myelination-driving master transcription factor Krox20 (also known as Egr2) through suppression of myelination inhibitory Notch signaling. As let-7 is also highly expressed in CNS, it remains unknown if let-7 is also responsible for CNS myelinations [80].

## 5. MicroRNAs and Intrinsic Determinants of Axon Regeneration

Although the glia scar and extrinsic inhibitory elements are the most important barrier of axon regeneration, most axons still cannot regenerate after even eliminating glia scar and improving the hostile environment. There are some intrinsic factors that have been shown to be playing pivotal roles in axon regeneration in CNS. The Krüppel-like family of transcription factors (KLFs) are a set of zinc finger DNA-binding proteins that regulate gene expression. Several KLFs family members have been shown to be playing pivotal roles in axon regeneration. KLF6 and KLF7 have the opposite functions in neurite growth while deletion of KLF4 improves neurite growth in vitro and in optic nerve regeneration after crush in vivo [81–83]. Conditioned deletion of PTEN and/or SOCS3 genes in mice can significantly improve the intrinsic axon regeneration potential and promote the axon regrowth into lesion sites [84]. In addition, a series of signaling alternations, including p53, calcium, MAPK, JAK/STAT, and mTOR pathway, also have been detected after injury [53, 54]. Obvious axon regeneration has been obtained by modulating different injury-induced signaling pathways, such as KLFs, PTEN, GSK3*β*, mTOR, STAT3, b-RAF, SOX11, DLK-1, cAMP, RhoA, and SOCS3 [62, 85–88].

Emerging evidences demonstrate that there is a close relationship between microRNAs and intrinsic determinants of axon regeneration. For example, miR-133b, which is specifically expressed in mammalian midbrain dopaminergic neurons (DNs) and is deficient in midbrain tissue from patients with Parkinson's disease [89], has been proved as an important determinant in spinal cord regeneration in adult zebra fish by directly reducing RhoA protein levels [90]. miR-133b has also been shown to be promoting neurite outgrowth in the primary cortical neurons and PC12 cells [91]. miR-133b increases axon growth and attenuates axon growth restrictions from CSPG in PCNs via ERK1/2 and PI3K/Akt signaling pathway by suppressing RhoA [91].

There are several other microRNAs which have been identified as important regulators of axon regeneration, and some of them have crosstalk with the critical genes involving axon regeneration after CNS injury and degeneration. In our recently published research, we found that miR-26a promotes axon regeneration by suppressing GSK3*β* expression in mammals [92]. In retinal ganglion cells, miR-30b has been proved to promote axon outgrowth by inhibiting the expression of semaphorin 3A (Sema3A), which is a major inhibitory factor of optic nerve (ON) regeneration after injury [93]. miR-132, one of the brain enriched microRNAs, is abundant in developing axons relative to mature axons, and it can promote axon extension of cultured DRG axons through repressing the Ras GTPase activator Rasa1, a novel target in neuronal function [94]. Interestingly, miR-132 is the target of the transcription factor, cAMP-response element binding protein (CREB). miR-132 regulates neuronal morphogenesis by decreasing levels of the GTPase-activating protein, p250GAP [95]. miR-431, one of nerve injury-induced microRNAs, stimulates regenerative axon growth by silencing Kremen1, an antagonist of Wnt/beta-catenin signaling [96]. Both the gain-of-function of miR-431 and knockdown of Kremen1 significantly enhance axon outgrowth in murine dorsal root ganglion neuronal cultures. More recently, miR-431 is also found to regulate motor neuron neurite length by targeting several molecules, such as chondrolectin and Kif3B, previously identified to play a role in motor neuron axon outgrowth [97].

miR-124, the most abundant and well-conserved brain-specific microRNA, is involved in regulating neurite elongation by targeting ROCK1, KLFs, and STAT3 [98]. STAT3 and KLFs can be regulated by miR-185, miR-19b, let-7, miR-22, miR-203, miR-93, miR-10b, miR-337, miR-145, and so on [99–103], and some of these microRNAs are dysregulated after SCI (Table 1). In contrast, KLF4 can directly upregulate miR-203 to promote cell senescence [104]. Furthermore, a mutual negative feedback loop between miR-138 and SIRT1 exists in the process of axon regeneration following peripheral nerve injury [105]. In the future, more investigations are still needed to fully reveal the complex regulatory network involving multiple genetic and epigenetic factors in axon regeneration.

## 6. Therapeutic Potentials of MicroRNAs for the Treatment of SCI

SCI is a devastating disease and often leads to severe disability. Several therapeutic strategies have been proposed to support axon regeneration and neurologic function rehabilitation, such as conditioned lesion, cell transplantation, epigenetic regulation, artificial scaffold transplantation, and gene therapy [106, 107]. However, there are still no effective medications currently available for treatment of SCI. As microRNAs have the ability of fine-tuning the expression of multiple targets and they usually have tissue-specific expression patterns, it is easier to design tissue-specific gene targeting tools which have great therapeutic potentials for treatment of SCI [108]. As discussed above, microRNAs compose a complex network with both intrinsic and extrinsic determinants of axon regeneration after SCI. For example, miR-199a, miR-124, and miR-133b regulate neurite outgrowth by targeting intrinsic factors PTEN, POCK1, and RhoA, respectively (Table 2). While miR-21, miR-145, miR-146, miR-181, miR-125b, and miR-486 play various roles in astrocytosis and astrogliosis, let-7, miR-9, miR-23, miR-138, miR-219, and miR-146 promote myelination after SCI (Table 2). Manipulating the expression of microRNAs in injury sites might not only neutralize the local environment to make it more permissive for nerve regeneration, but also activate intrinsic genes in neurons that contribute to axon regeneration.

In principle, the functional recovery could be achieved by intrinsic or extrinsic promoting of axonal regrowth: the regeneration of lesioned axons which will reconnect to their original targets and the sprouting of spared axons that form new circuits and compensate for the lost function. Therefore, the important approaches in spinal cord are to manipulate and neutralize the local environment, such as modulating glial scar formation and oligodendrogenesis, to make it more permissive for nerve regeneration, although recreation of the growth-promoting environment after nerve injuries remains challenging [6].

So far, there are no clinical trials on microRNA-based treatment for SCI in humans. MicroRNA-based therapies could involve the administration of a specific microRNA mimic to downregulate target genes or antisense probes for the blocking of certain microRNAs to increase the expression of target genes in injury sites. In recent years, microRNA delivery technology development is growing rapidly and gives us high expectation for microRNAs as therapeutics. Virus-based microRNA delivery technology has shed light on the development of microRNAs delivery systems for SCI treatment. It has been demonstrated that serotype 9 of AAV (AAV9) vectors shows the highest tropism for neural tissue and can cross the blood-brain barrier, and the authors have shown that intrathecal delivery of AAV9 yields relatively high gene transduction into dorsal root ganglia or spinal cord [109]. More importantly, intracardiac injections of tyrosine-mutant pseudotype AAV9/3 vectors result in extensive and widespread transgene expression in the spinal cords of adult mice, which suggests that tyrosine-mutant AAV9/3 vectors may be effective vehicles for delivery of therapeutic genes, including microRNAs, into the spinal cord for treating diseases [110]. However, one of the most important challenges in this field is the need for the development of noninvasive approaches to delivering ncRNA modulators, such as anti-microRNAs or microRNA mimics, into the spinal cord without significant off-target effects [111].

## 7. Conclusion and Perspective

SCI is a devastating disease and often leads to severe disability. Unfortunately, there is still no clinical treatment currently available that can achieve the same outcome as in animals. The regeneration capacity of nerve tissues after SCI is very limited. This poor regeneration is mainly contributed by both the hostile microenvironment at the injured sites and the limited axon regrowth potential of adult CNS neurons. MicroRNAs take part in a series of pathophysiological processes and play pivotal roles in the process of maintaining homeostasis following SCI. MicroRNAs can regulate inflammation, eliminate myelin debris, suppress excessive astrogliosis to improve hostile microenvironment, and directly or indirectly modulate gene expression to promote intrinsic axon regeneration ability. As microRNAs are small molecules that can be easily delivered, usually have tissue-specific expression properties, and fine-tune the expression of multiple genes at a time, they have great therapeutic potentials for SCI gene therapy. To date, we still know very little about the molecular mechanisms underlying axon regeneration and the pathogenesis of SCI, and the complete regulatory network involving microRNAs and other genetic and epigenetic factors is largely unknown. In addition, great efforts should also be made to examine the therapeutic potentials of microRNAs and to develop effective microRNA-based treatment approaches for SCI.

## Figures and Tables

**Table 1:** Differentiated expression of microRNAs in different spinal cord injury models.

Expression patterns	Spinal cord injury model		Reference
	Ischemia-reperfusion injury	Contusion injury	
Upregulation	miR-204, miR-365, miR-323, miR-672, miR-760-5p, miR-376b-5p, miR-369-5p, miR-133a, miR-505, miR-466d, miR-132, miR-665, miR-463	miR-1, miR-15b, miR-20a, miR-20b-5p, miR-21, miR-30a, miR-31, miR-92a, miR-92b, miR-93, miR-98, miR-106b, miR-145, miR-146b, miR-152, miR-199a-3p, miR-203, miR-206, miR-214, miR-221, miR-223, miR-290, miR-333, miR-374, miR-378, miR-672, miR-674-5p, miR-872, miR-17, miR-146a, miR-124, miR-486	[15, 23, 24, 112, 113]

Downregulation	miR-210, miR-146a, miR-199a-3p	miR-30b-5p, miR-30c, miR-30d, miR-34a, miR-129, miR-137, miR-138, miR-219-2-3p, miR-219-5p, miR-323, miR-325-3p, miR-338, miR-379, miR-384-5p, miR-495, miR-543, miR-708, miR-125-3p, miR-126, miR-let-7b, miR-129-1, miR-129-2, miR-129-3p, miR-342	[21, 36, 37, 113, 114]

Early: upregulation; late: downregulation^*∗*^		miR-99a, miR-100, miR-103, miR-107, miR-124, miR-127, miR-128, miR-154, miR-181a, miR-434, miR-487b, miR-124a, miR-133a, miR-133b, miR-45	[15, 23, 90]

^**∗**^MicroRNAs are significantly upregulated at 4 hours and then downregulated at 7 days after SCI.

**Table 2:** The function of miRNAs that might have therapeutic potential for the treatment of SCI.

Cell type	miRNA	Target	Function	Experiment	Reference
Neuron	miR-199a	PTEN	Impeding neurite outgrowth	In vivo	[112]
	miR-124	ROCK1	Promoting neurite outgrowth	In vitro	[98]
	miR-133b	RhoA	Promoting neurite outgrowth	In vitro	[90]

Astrocytes	miR-21	Unidentified	Attenuating the astrocytes hypertrophic response to SCI	In vivo	[66]
	miR-145	BMP	Reducing astrocytic cell density at the lesion border of the injured spinal cord	In vivo	[39]
	miR-146	GFAP, c-Myc	Regulating inflammation response	In vitro	[70, 71]
	miR-181	SOD2	Reducing astrocyte death in vitro and infarct volume after stroke in vivo	In vivo and in vitro	[31, 60]
	miR-125b	CDKN2A	Promoting astrogliosis and defects in the cell cycle	In vivo	[72]
	miR-486	NeuroD6	Neuroprotection	In vivo	[115]

Oligodendrocyte	Let-7	Sox4, UHRF1b-p1	Promoting myelination	In vitro	[73]
	miR-9	PMP22	Promoting OLs differentiated	In vitro	[116]
	miR-23	LaminB1	Promoting oligodendrocyte development and myelination	In vitro	[78, 79]
	miR-138	Sox4	Promoting OL differentiation	In vitro	[73]
	miR-219	PDGFR-*α*, Sox6, FoxJ3, ZFP238	Promoting OPC proliferation	In vivo	[73, 75]
	miR-146	Unidentified	Promoting oligodendrogenesis	In vivo	[117]
